# Impact of provincial and national implementation strategies on P2Y12 inhibitor utilization for acute coronary syndrome in the elderly: an interrupted time series analysis from 2008 to 2018

**DOI:** 10.1186/s13012-021-01117-z

**Published:** 2021-04-21

**Authors:** Saurabh Gupta, Emilie P. Belley-Cote, Adam Eqbal, Charlotte McEwen, Ameen Basha, Nicole Wu, Joshua O. Cerasuolo, Shamir Mehta, Jon-David Schwalm, Richard P. Whitlock

**Affiliations:** 1 Division of Cardiac Surgery, Department of Surgery, McMaster University, Hamilton, Ontario Canada; 2 Department of Health Research Methods, Evidence and Impact, McMaster University, Hamilton, Ontario Canada; 3 Division of Cardiology, Department of Medicine, McMaster University, Hamilton, Ontario Canada; 4 Population Health Research Institute, Hamilton, Ontario Canada; 5 Faculty of Health Sciences, McMaster University, Hamilton, Ontario Canada; 6 Faculty of Sciences, McMaster University, Hamilton, Ontario Canada; 7 ICES McMaster, Faculty of Health Sciences, McMaster University, Hamilton, Ontario Canada; 8 David Braley Cardiac, Vascular and Stroke Research Institute, 237 Barton St. E, Hamilton, Ontario L8L 2X2 Canada

## Abstract

**Background:**

Guidelines recommend both acetylsalicylic acid and ticagrelor following acute coronary syndrome (ACS), but appropriate prescription practices lag. We analyzed the impact of government medication approval, national guideline updates, and publicly funded drug coverage plans on P2Y12 inhibitor utilization.

**Methods:**

Accessing provincial databases, we obtained data for elderly ACS patients in Ontario, Canada, between 2008 and 2018. Using interrupted-time series with descriptive statistics and segmented regression analysis, we evaluated types of P2Y12 inhibitors prescribed at discharge and changes to their utilization in patients managed with percutaneous intervention (PCI), coronary artery bypass grafting (CABG) or medically, following national antiplatelet therapy guidelines (by the Canadian Cardiovascular Society), ticagrelor’s national approval by Health Canada, and ticagrelor’s coverage by a publicly funded medication plan.

**Results:**

We included 114,142 patients (49.4%-PCI; mean age 75.71±6.94 and 62.3% male and 7.7%-CABG; mean age 74.11±5.63 and 73.5% male).

Among PCI patients, clopidogrel utilization declined monthly after 2010 national guidelines were published (*p*<0.0001) and within the first month after ticagrelor’s national approval by Health Canada (*p*=0.03). Among PCI patients, ticagrelor utilization increased within the first month (*p*<0.0001) and continued increasing monthly (*p*<0.0001) after its coverage by a publicly funded medication plan. Among PCI patients, clopidogrel utilization declined within the first month (*p*=0.003) and ticagrelor utilization increased monthly (*p*=0.05) after 2012 CCS guidelines.

Among CABG patients, ticagrelor’s coverage was associated with a monthly increase in its utilization (*p*<0.0001).

**Conclusion:**

National guideline updates and drug coverage by a publicly funded medication plan significantly improved P2Y12 inhibitor utilization. Barriers to appropriate antiplatelet therapy in the surgical population must be explored.

Contributions to the literature

- Significant gaps exist between evidence generation and implementation in cardiovascular health. Publishing new evidence rarely influences practitioners into adopting it.
- Using the knowledge-to-action framework, we discovered that policy changes—such as coverage by a publicly funded model—were essential in uptake of evidence.
- Personal beliefs around safety and efficacy, despite evidence and guidelines, continue to undermine implementation efforts. With help from facilitators, barriers to appropriate therapies must be addressed.
- Our findings contribute to already recognized gaps in the literature, including determining how and why evidence is used and who is most influenced.

## Introduction

Based on large, multi-center randomized controlled trials (RCTs), guidelines recommend dual antiplatelet therapy (DAPT) with acetylsalicylic acid (ASA) and a P2Y12 inhibitor for 12 months after an acute coronary syndrome (ACS), regardless of management strategy: medical management, percutaneous intervention (PCI), or coronary artery bypass grafting (CABG) [1–10]. Specifically, the 2010 Canadian Cardiovascular Society (CCS) Antiplatelet Therapy Guidelines recommended DAPT with ticagrelor or clopidogrel and ASA for all ACS patients. Soon after, a focused update was released in 2013, exclusively recommending ticagrelor and ASA for all ACS patients [10, 11]. Despite these recommendations and available evidence, appropriate DAPT use remains variable. For example, the ACS Reflective Program—a multicenter national observational registry conducted from 2011 to 2013—found that clopidogrel remained the most common prescribed P2Y12 inhibitor across Canada at a rate of 82%, compared to only 9% for ticagrelor. Further, the prospective Canadian Observational Antiplatelet Study (COAPT) demonstrated that despite RCTs and guideline recommendations, only a minority of ACS patients were discharged on ticagrelor (11.1%) or prasugrel (5.7%), compared to clopidogrel, after PCI [12, 13]. DAPT is particularly underused in patients undergoing CABG. In a cohort of patients with ACS, CABG was an independent predictor for DAPT underutilization (odds ratio [OR] 0.09, 95%CI 0.05–0.14) [14–16].

Suboptimal evidence implementation is not unique to antiplatelet therapy and ACS patients. Glynn et al. reviewed random patient samples from 12 metropolitans in the United States (U.S.) and found that less than 55% received guideline-recommended care [17]. More specific to cardiovascular health, a review of care gaps (defined as under-utilization or under-dosing of proven treatments) found that only 34 to 60% of eligible outpatients in Europe, Canada, and U.S. received appropriate medications for heart failure, coronary artery disease, or atrial fibrillation [18]. As such, implementation science strategies are paramount in improving healthcare delivery and quality of care.

To address the knowledge-implementation gap around antiplatelet therapy and ACS, we used provincial administrative data to examine how—if at all—government approval, guideline recommendations, and publicly funded medication plan coverage impacted P2Y12 inhibitor utilization among the elderly ACS patients managed medically, with PCI or CABG.

## Methods

### Study design

To examine the impact of specific provincial and national implementation strategies on P2Y12 inhibitor utilization, the authors conducted an interrupted time series design (with descriptive statistics and regression analysis) on population-level data, which was aggregated monthly. This design was felt to be most appropriate given the clearly defined points in time when the various policy and guideline changes were enacted in Ontario. Details around use of interrupted time series to create an ecological-level analysis to evaluate quality improvement in health care or public health interventions were adapted from Bernal et al. and Penfold et al. [19, 20]. An interrupted time series analysis allows us to control for underlying trends that may already exist in the population, including cumulative impacts of pre-existing policies. As such, we can model the impact our intervention has on the population, at time of implementation.

### Reporting guidelines

The authors used the “Standards for Reporting Implementation Studies: the StaRI checklist for completion” by Pinnock et al. to report methods, results, and discussion [21].

### Study population

We included all patients 65 years of age or older who presented with an ACS and were subsequently referred for coronary angiogram in the province of Ontario, Canada from October 1, 2008 (chosen as such due to availability of complete Cardiac Care Network (CCN) datasets from then onwards) to March 31, 2018. Ontario includes 20 cardiac centers, which provide care to 14.5 million inhabitants. Apart from universal healthcare, the provincial government provides a publicly funded medication plan for those 65 years or older (Ontario Drugs Benefits (ODB)).

### Databases and cohort definition

We obtained data on baseline characteristics and prescriptions filled from five databases linked at the Institute for Clinical Evaluation Sciences (ICES)—CorHealth, Ontario Drug Benefit (ODB) database, Discharge Abstract Database (DAD), and Same-Day Surgery Database (SDS) from Canadian Institute for Health Information (CIHI), National Ambulatory Care Reporting System (NACRS), and Registered Persons Database (RPDB). These datasets were linked using unique encoded identifiers and analyzed at ICES. They were used to analyze health outcomes as individuals can be tracked over time [22–25]. Available data on admission for ACS, patient characteristics, and management strategies were captured retrospectively from the CCN (CorHealth) database.

Using variables from CCN, we applied the following cohort exclusions: aged younger than 65 years or older than 105 years (which was derived from RPDB), did not have a coronary angiogram within 3 months of ACS, primary reason for cardiac surgery referral was not coronary disease, died prior to discharge from facility performing coronary angiogram, normal anatomy on angiogram, or non-obstructive coronary artery disease. Definitions for all other clinical variables are available on the CCN website (https://www.corhealthontario.ca/data-&-reporting/data-collection-&-access/CCN-Registries-Data-Entry-Reference-Manual-&-Data-Standards-Document-Updated-September-2017.pdf). We identified patients undergoing PCI or CABG from DAD and SDS databases by ICD-10 codes: 1.IJ.50, 1.IJ.54 or 1.IJ.57 for PCI and 1.IJ.76 for CABG.

### Outcomes and interventions

We tracked the number of P2Y12 inhibitor dispensations in Ontario Drug Benefits database within 14 days (per Ontario Primary Care Medication Reconciliation Guide, medications should be reconciled within 14 days from hospital discharge) of discharge from index hospitalization (that is, when the ACS occurred). We collected data on the proportion of dispensed P2Y12 inhibitors (eligible agents included ticagrelor, clopidogrel, and prasugrel) and included any patient with at least one dispensation. Of note, we did assume that patients taking either one of these antiplatelet agents were also taking ASA, since it is available over the counter and cannot be tracked by ODB.

Our outcome of interest was the change, over time, in P2Y12 inhibitor utilization after specific implementation strategies (interventions). The implementation strategies were enacted across the province of Ontario. They were intended to target physicians, surgeons, and pharmacists who would help manage patients suffering an ACS. These interventions were decided by discussion among the authors and feedback from reviewers:

1. Approval of ticagrelor by Health Canada in June 2011. The approval was marketed across the province and nation via press releases, discussion of the “Platelet Inhibition and Patient Outcomes” (PLATO) trial, and distribution of “Brilinta Saving Cards” to eligible patients;
2. Changes to the “Use of Antiplatelet Therapy in the Outpatient Setting: CCS Guidelines” (herein called CCS Antiplatelet Therapy Guidelines) in May 2011 and November 2013. The changes to the national guidelines were communicated by announcements at the annual Canadian Cardiovascular Congress, via “News Release” by Heart & Stroke™ Canada, along with using an “E-Guideline” platform creating summary videos;
3. Publicly funded medication plan (ODB) coverage of ticagrelor for ACS patients in April 2013. List of medications funded through ODB are directly available to pharmacists and certain physicians through the Ontario Ministry of Health and Long-Term Care’s Health Network System. As well, ODB approval of new medications is announced through an e-mail system called, “OneMail”.

### Statistical analysis

We created an interrupted time series with the data aggregated at the population-level monthly. The dependent variable was the proportion of patients dispensed a P2Y12 inhibitor within 14 days of hospital discharge. Our autoregressive model included the following covariates:

1. Intervention—binary indicator denoting 0 during pre-intervention period and 1 during post-intervention period;
2. Time (in months)—ordinal indicator denoting months since start of study;
3. Time since intervention (in months)—ordinal indicator denoting months since time interruption (i.e., implementation of intervention);
4. Autoregressive processes (if applicable).

We also incorporated lag periods in our interrupted time series; they included the month of intervention and 3 months after (to allow for uptake of intervention into clinical practice). All data points enclosed in the lag period were excluded from analysis. Given the availability of data, this allowed us to increase the number of timepoints before and after interventions, providing sufficient statistical power.

To build the model, we initially added 13 autoregressive parameters because the datasets were aggregated by month—13 processes allow the models to detect and adjust for seasonality. In a backward stepwise fashion, autoregressive parameters were removed until all autoregressive parameters remained statistically significant. Presence of autoregression (or autocorrelation) was assessed using Durbin-Watson statistic [26]. Presence of seasonality (stationarity) was assessed using Dickey-Fuller statistic [27].

The impact of interventions was also stratified by management strategy of each ACS patient—with PCI, with CABG, or medically.

The output and data analysis for this study was generated using SAS software.

### Ethics approval

The study was approved by the Hamilton Integrated Research Ethics Board (HiREB #4196). ICES is an independent, non-profit research institute whose legal status under Ontario’s health information privacy law allows it to collect and analyze health care and demographic data, without consent, for health system evaluation and improvement.

## Results

### Population characteristics

**Fig. 1:**
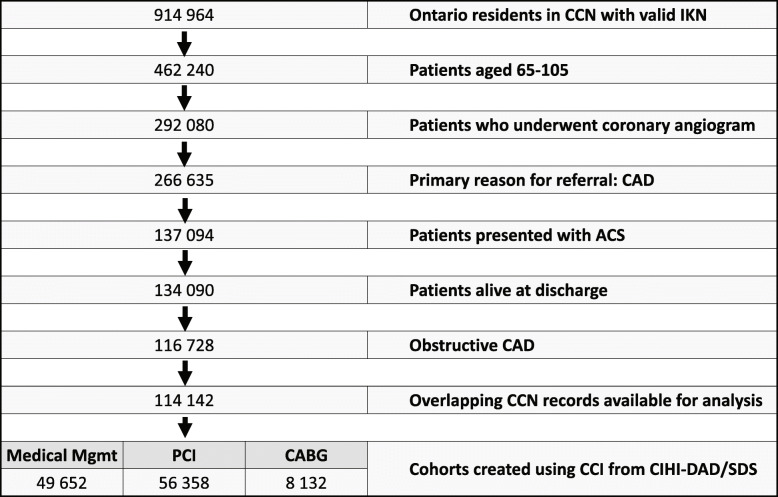
Study flow diagram demonstrating development of cohorts

Figure 1 represents the study flow diagram. We identified a total of 114,142 elderly patients and present their baseline characteristics in Table 1. PCI was performed in 49.4% of patients (mean age, 75.71 ± 6.94, 62.3% male), CABG in 7.7% of patients (mean age, 74.11 ± 5.63; 73.5% male), and 42.9% were managed medically (mean age, 75.71 ± 6.50; 62.9% male). Medically managed patients were more likely to have undergone previous interventions (PCI and/or CABG) and had more comorbidities with a higher proportion presenting with congestive heart failure (CHF), chronic obstructive pulmonary obstruction (COPD), cerebrovascular disease (CVD), and peripheral vascular disease (PVD). Further, medically managed patients were more likely to already be on antithrombotic medications before their ACS. At the beginning of our accrual period, the proportion of ACS patients prescribed P2Y12 inhibitors at discharge was 73.4% in the PCI group, 11.4% in the CABG group, and 31.4% in the medically managed group. As of March 2018, the proportion had increased to 86.9% (*p*<0.001) for patients undergoing PCI, 46.5% (*p*<0.001) for those undergoing CABG, and 32.3% (*p*=0.775) for those managed medically.

**Table 1:** Characteristics of ACS patients stratified by management strategy

Characteristic	Medical management *n*=49,652	PCI *n*=56,358	CABG *n*=8,132	*p* value
Age*	75.71 ± 6.50	75.71 ± 6.94	74.11 ± 5.63	<.001
Male sex (%male)	30,742 (61.9%)	35,137 (62.3%)	5973 (73.5%)	<.001
Current smoker	6521 (13.1%)	8010 (14.2%)	1272 (15.6%)	NS
Previous smoker	17,301 (34.8%)	16,532 (29.3%)	2866 (35.2%)	<.001
COPD	5276 (10.6%)	4552 (8.1%)	598 (7.4%)	<.001
CVD	5723 (11.5%)	5240 (9.3%)	809 (9.9%)	<.001
CHF	7157 (14.4%)	4962 (8.8%)	812 (10.0%)	<.001
Diabetes	18,193 (36.6%)	16,737 (29.7%)	2855 (35.1%)	<.001
Hyperlipidemia	37,018 (74.6%)	34,964 (62.0%)	5418 (66.6%)	<.001
Hypertension	39,566 (79.7%)	40,309 (71.5%)	6025 (74.1%)	<.001
History of MI	18,988 (38.2%)	15,950 (28.3%)	1947 (23.9%)	<.001
PVD	5583 (11.2%)	4,533 (8.0%)	839 (10.3%)	<.001
Renal disease	261 (0.5%)	215 (0.4%)	31 (0.4%)	NS
Previous CABG	10,643 (21.4%)	8042 (14.3%)	251 (3.1%)	<.001
Previous PCI	11,722 (23.6%)	12,284 (21.8%)	977 (12.0%)	<.001
Previous antithrombotic				
Dabigatran	744 (1.5%)	630 (1.1%)	58 (0.1%)	<.001
Apixaban	779 (1.6%)	795 (1.4%)	66 (0.8%)	<.001
Rivaroxaban	992 (2.0%)	1,156 (2.1%)	116 (1.4%)	<.001
Warfarin	4,894 (9.9%)	3,700 (6.6%)	396 (4.9%)	<.001
Prasugrel	21 (0.0%)	36 (0.1%)	0 (0.0%)	NS
Clopidogrel	11,575 (23.3%)	10,614 (18.8%)	1029 (12.7%)	<.001
Ticagrelor	589 (1.2%)	992 (1.8%)	45 (0.6%)	<.001

*Mean ± SD

Abbreviations: *COPD* Chronic obstructive pulmonary disease, *CVD* Cerebrovascular disease, *CHF* Congestive heart failure, *MI* Myocardial infarction, *PVD* Peripheral vascular disease, *CABG* Coronary artery bypass grafting surgery, *PCI* Percutaneous coronary intervention

The baseline characteristics, including medical co-morbidities and previous antithrombotic use, were measured when patient presented with their ACS

While we evaluated prasugrel utilization independently, the number of patients discharged on prasugrel was extremely low, and we were unable to conduct a robust analysis; the models did not converge and could not be interpreted.

**Fig. 2:**
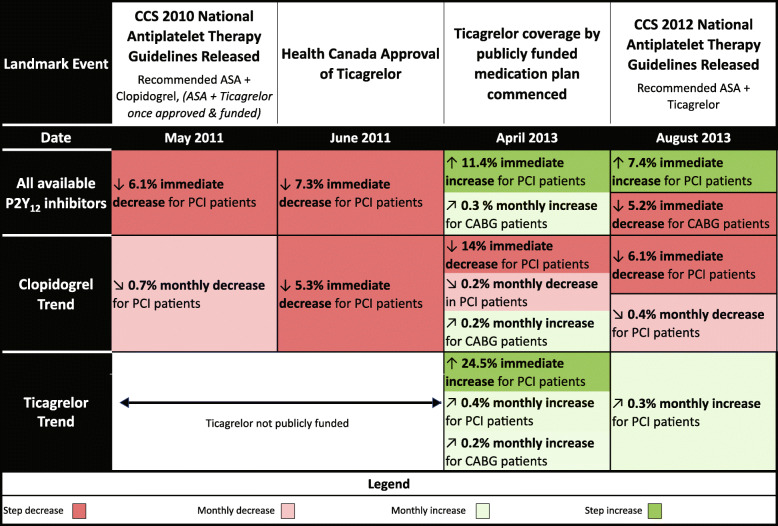
Drug coverage by a publicly funded medication plan and guideline updates had significant impact on P_2_Y_12_ inhibitor prescription practices

Figure 2—our central image—reviews all key findings for PCI and CABG patients as detailed below.

### P2Y12 inhibitor utilization and 2010 CCS antiplatelet therapy guidelines *(published in 2011)* (Fig. 3)

We could not evaluate the impact of the 2010 CCS Antiplatelet Therapy Guidelines on ticagrelor because it was not yet approved by ODB.

**Fig. 3:**
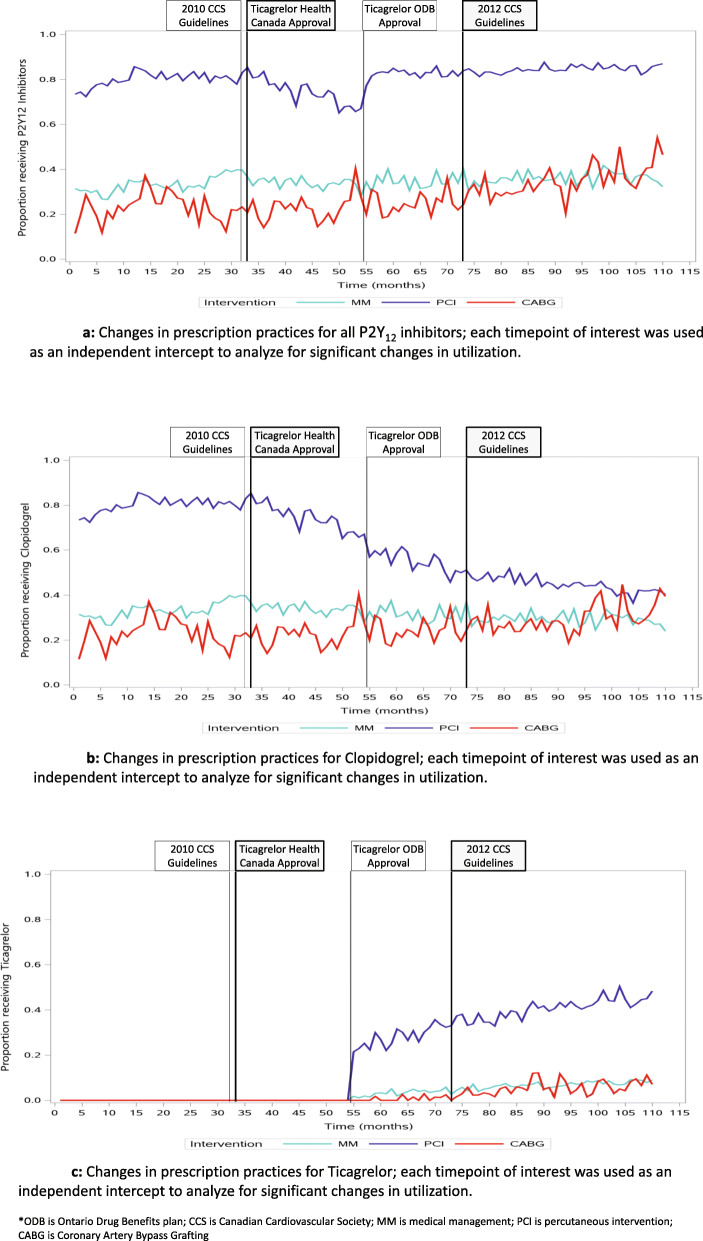
**a** Changes in utilization of all available P2Y12 inhibitors from 2008 to 2018. Each time point of interest was used as an intercept to analyze for significant changes in practice patterns. **b** Changes in Clopidogrel utilization from 2008 to 2018. Each time point of interest was used as an intercept to analyze for significant changes in practice patterns. **c** Changes in Ticagrelor utilization from 2008 to 2018. Each time point of interest was used as an intercept to analyze for significant changes in practice patterns. MM medically managed, PCI percutaneous intervention, and CABG coronary artery bypass grafting

### PCI patients

The publication of the 2010 CCS Antiplatelet Therapy Guidelines in May 2011 was associated with a significant decline of 6.1% (*p*=0.003) in utilization of all available P2Y12 inhibitors within the first month. The trend continued, but not significantly, in the months after. Individual clopidogrel utilization also declined within the first month after guideline updates, albeit not significantly. Clopidogrel utilization, however, declined significantly by 0.7% (*p*<0.0001) every month thereafter.

### CABG patients

The publication of 2010 CCS Antiplatelet Therapy Guidelines in May 2011 was associated with a decline in utilization of all available P2Y12 inhibitors within the first month and every month thereafter; however, this was not significant. Clopidogrel utilization demonstrated similar trends, which were also not significant.

### Medically managed patients

The publication of 2010 CCS Antiplatelet Therapy Guidelines was associated with a significant decline of 3.8% (*p*=0.002) in utilization of all available P2Y12 inhibitors within the first month, followed by a significant decline of 0.2% (*p*=0.002) every month after. Individually, the guideline updates also impacted clopidogrel utilization within the first month and were associated with a significant decline of 2.5% (*p*=0.02), followed by a significant decline of 0.3% (*p*<0.0001) every month after.

### P2Y12 inhibitor utilization and ticagrelor Health Canada approval *(in 2011)* (Fig. 3)

We could not evaluate the impact of ticagrelor’s Health Canada approval on ticagrelor prescriptions itself as it was not approved by ODB yet.

### PCI patients

The approval of ticagrelor by Health Canada in June 2011 was associated with a significant decline of 7.3% (*p*<0.002) in utilization of all available P2Y12 inhibitors within the first month, and while the trend continued monthly thereafter, it was not significant. Utilization of clopidogrel declined by 5.3% (*p*=0.03) within the first month and continued to decline every month thereafter, but not significantly.

### CABG patients

The approval of ticagrelor by Health Canada in June 2011 demonstrated a decline in utilization of all available P2Y12 inhibitors, but this was not significant. The trend continued every month thereafter, without reaching statistical significance. Clopidogrel utilization showed similar trends, which also failed to reach statistical significance.

### Medically managed patients

The approval of ticagrelor by Health Canada in June 2011 was associated with a significant decline of 4.3% (*p*<0.001) in utilization of all available P2Y12 inhibitors within the first month and continued to significantly decline monthly thereafter by 0.2% (*p*<0.001). Clopidogrel utilization also declined by 3.2% (*p*=0.003) within the first month and continued to significantly decline every month thereafter by 0.3% (*p*<0.001).

### P2Y12 inhibitor utilization and coverage of ticagrelor by the publicly funded medication plan *(in 2012)* (Fig. 3)

#### PCI patients

Ticagrelor’s coverage by the publicly funded medication plan in August 2013 was associated with an increase of 11.4% (*p*<0.0001) in utilization of all available P2Y12 inhibitors within the first month after coverage began, and while the trend continued every month after, it was not significant. The approval was associated with a significant decline of 14% (*p*<0.0001) in clopidogrel utilization, followed by a decline of 0.2% (*p*=0.03) every month thereafter. Meanwhile, ticagrelor utilization increased by 24.5% (*p*<0.0001) within the first month and continued to increase by 0.4% every month (*p*<0.0001) thereafter.

#### CABG patients

Ticagrelor’s coverage by the publicly funded medication plan in August 2013 was not associated with a significant change in utilization of all available P2Y12 inhibitors, or clopidogrel and ticagrelor individually, within the first month. However, after, there was a significant increase of 0.3% (*p*=0.0001) every month in utilization of all available P2Y12 inhibitors. With the coverage, clopidogrel utilization also increased every month after by 0.2% (*p*=0.04). Meanwhile, ticagrelor utilization also increased by 0.2% (*p*<0.0001) every month after its approval.

### Medically managed patients

Ticagrelor’s approval by the publicly funded medication plan in August 2013 was not associated with a significant impact on utilization of all available P2Y12 inhibitors. Individually, the approval was associated with a significant decline of 0.1% (*p*=0.005) in clopidogrel utilization every month after. Meanwhile, ticagrelor utilization demonstrated a significant increase of 2.2% (*p*<0.0001) within the first month after its approval and continued to significantly increase by 0.12% (*p*<0.0001) every month after.

### P2Y12 inhibitor utilization and 2012 CCS Antiplatelet Therapy Guidelines *(published in 2013)* (Fig. 3)

#### PCI patients

The publication of 2012 CCS Antiplatelet Therapy Guidelines in August 2013 was associated with a significant increase of 7.4% (*p*=0.0004) in utilization of all available P2Y12 inhibitors within the first month after publication. The trend continued in the subsequent months but was not significant. The updated guidelines were associated with a decline of 6.1% (*p*=0.003) in clopidogrel utilization within the first month after publication, followed by a significant decline of 0.4% (*p*=0.002) every month thereafter. Meanwhile, ticagrelor utilization demonstrated an increase within the first month, albeit not significant. Thereafter, ticagrelor utilization significantly increased by 0.3% (*p*=0.05) every month.

#### CABG patients

With the publication of 2012 CCS Antiplatelet Therapy Guidelines in August 2013, utilization of all available P2Y12 inhibitors decreased by 5.2% (*p*=0.05) within the first month but did not demonstrate a significant trend every month thereafter. While clopidogrel utilization also declined within the first month, and monthly afterwards, the change was not significant. Similarly, while ticagrelor utilization increased every month after guideline publication, the change was not significant.

#### Medically managed patients

The publication of 2012 CCS Antiplatelet Therapy Guidelines was not associated with a significant change in utilization of all available P2Y12 inhibitors within the first month or after. Individually, the guideline updates were also not associated with a significant change in clopidogrel utilization. After the 2012 guideline updates, ticagrelor utilization, however, demonstrated a significant increase of 1.7% (*p*<0.0001) within the first month. This trend continued every month after but was not significant.

## Discussion

We analyzed P2Y12 inhibitor utilization data on 114,142 elderly ACS patients and identified three key evidence-practice gaps. First, the uptake of evidence is slow and although the proportion of patients receiving P2Y12 inhibitors after an ACS has improved in the last decade, the proportion of patients receiving guideline-directed therapy with ticagrelor remains low. Second, DAPT use is suboptimal for patients who undergo CABG. Third, and perhaps most importantly, policies have the biggest influence on changing practice. Drug coverage by a publicly funded medication plan and guideline updates had the largest impact on appropriate medication utilization.

DAPT has proven benefit for ACS patients undergoing medical management, PCI or CABG [6, 7]. An RCT of 18,624 patients randomized to ASA and ticagrelor demonstrated that the latter reduced the primary composite outcome of vascular mortality, myocardial ischemia (MI), or cerebrovascular accidents (CVA)—9.8% with ASA and ticagrelor vs 11.75% with ASA and clopidogrel (HR 0.84; 95%CI 0.77–0.92) at 12 months [6]. Previously, Yusuf *et al*. compared DAPT with ASA and clopidogrel with ASA monotherapy and demonstrated that a composite of death from cardiovascular causes, non-fatal MI or stroke occurred in 9.3% of patients receiving DAPT and in 11.4% of patients receiving ASA only (RR 0.80; 95% CI 0.72–0.90; *p*<0.001) [7]. The results of CABG subgroups from both above trials were also consistent with above findings, demonstrating a reduction in major adverse cardiovascular events (MACE) when using more potent antiplatelet therapy regimens [28, 29]. Recently, Zhao et al. randomized 500 CABG patients to 1:1:1 (ticagrelor and ASA: ticagrelor monotherapy: ASA monotherapy) and reported a statistically significant increase in vein graft patency when comparing DAPT with ticagrelor and ASA to ASA monotherapy alone (12.2% [95% CI, 5.2% to 19.2%]; *p*<0.001) [9]. In a recent network meta-analysis, Gupta et al. studied the safety and efficacy of various antiplatelet regimens in 15,511 CABG patients and reported that DAPT with ASA and ticagrelor, compared to ASA monotherapy, reduced SVG stenosis (OR 0.40; 95% credible interval (CrI) 0.21, 0.74), mortality (OR 0.52; 95% CrI 0.30, 0.87) and MACE (OR 0.63; 95% CrI 0.44, 0.91) [30, 31]. As such, DAPT is an evidence-based and guideline-recommended treatment for patients with ACS regardless of the management approach.

Despite this, P2Y12 inhibitor prescription practices lag globally; our data corroborate this. We demonstrated that at end of our accrual window in March 2018, only 86.9% of PCI patients were discharged with P2Y12 inhibitors. This is not dissimilar to the previously published real-world data; Schwalm et al. reported a 92% compliance in P2Y12 inhibitor prescriptions after PCI in a cluster RCT, while and Turgeon et al. demonstrated a 80.5% compliance rate in their cohort of 13,897 ACS patients treated with PCI in Alberta from 2012 to 2016 [32, 33]. Esposti et al. analyzed discharge prescriptions of 1882 ACS patients in Italy. Of the 83% of patients who were discharged on any antiplatelet therapy, 57% were prescribed ASA and clopidogrel and only 0.1% of patients were discharged on ASA and ticagrelor [34]. DAPT is even further underutilized in ACS patients who undergo CABG compared to PCI. In a retrospective study of 8939 ACS patients in Australia, CABG was an independent predictor for DAPT underutilization; OR 0.09, 95%CI 0.05–0.14 [14]. A recent survey evaluating the practice patterns of 75 Canadian cardiac surgeons around postoperative antiplatelet management found that only 45% of cardiac surgeons would restart DAPT in patients who suffered a recent ACS. Interestingly, they were more likely to initiate DAPT in patients who had previous stents to vessels that were not bypassed, required endarterectomy, or suffered a peri-operative MI. Respondents (81%) were more concerned with preventing bleeding than recurrent ischemic events [35].

While underutilization of ticagrelor post-ACS in our cohort may appear concerning, we must note that our population is elderly, with a mean age over 74, and use of clopidogrel may be justified. Recently, the POPular AGE Trial (Clopidogrel versus ticagrelor or prasugrel in patients aged 70 years or older with non-ST-elevation acute coronary syndrome) demonstrated that ticagrelor was prematurely discontinued in 47% of patients (due to bleeding and dyspnea) compared to clopidogrel, which was only discontinued in 22% of patients. The authors also concluded that clopidogrel was an appropriate alternative to ticagrelor among ACS patients aged 70 years or older [36]. Within Canada, the Alberta Provincial Project for Outcome Assessment in Coronary Heart Disease registry evaluated clopidogrel versus ticagrelor prescriptions and outcomes in ACS patients who were discharged after a PCI. In their cohort of 11,185 PCI patients, compared with clopidogrel, ticagrelor was not associated with lower risk of MACE (aHR 0.97; 95% CI 0.85–1.10), but was associated with an increased risk of major bleeding (aHR 1.51; 95% CI 1.29–1.78) and dyspnea (aHR 1.98; 95% CI 1.47–2.65) [33]. These findings introduce a nuance to bleeding and dyspneic risk with ticagrelor, which seems particularly pronounced among the elderly, potentially explaining why clopidogrel remains so widely used in our cohort.

Additionally, it should be noted that evidence for use of DAPT after CABG has significant limitations—RCTs are small or are sub-studies of larger RCTs. Furthermore, there is significant population heterogeneity within the RCTs on whether patients underwent off-pump CABG (OPCAB) or CABG with cardiopulmonary bypass. In fact, a large majority of patients randomized in the two trials demonstrating DAPT superiority over ASA monotherapy underwent OPCAB [9, 37]. The available evidence does not evaluate the use of DAPT in patients undergoing multiple arterial grafting, which are associated with significantly better patency rates and clinical outcomes compared with vein grafts and the effect DAPT has on the latter may not apply to arterial grafts [38, 39]. Given the limitation of existing evidence, physicians and surgeons are more concerned with post-procedural bleeding risk, lack of dedicated robust evidence in CABG patients.

Given the above findings and real-world data, the discordance between guidelines and practice is likely multifactorial. The underutilization of ticagrelor may be due to some of the barriers to physician adherence discussed by Cabana et al. Specifically, with ever-expanding research, physicians may find it challenging to keep abreast with updated guidelines or apply them due to previous practice inertia. While lack of familiarity with ticagrelor (pharmacology and evidence around it) or external barriers (perceived issues with costs and drug coverage) may play a role, the most likely factors are likely due to lack of outcome expectancy (belief in efficacy) and lack of agreement (belief in safety, especially fear of increased bleeding, and belief in strength of recommendations) [40]. In fact, the fear of post-procedural bleeding may also explain why all P2Y12 inhibitor utilization among CABG patients declined significantly after the 2012 CCS Antiplatelet Therapy Guidelines were published. Despite strong recommendations for ASA and ticagrelor, surgeons were likely not convinced of the efficacy and safety in this population [12].

Our observation that provincial coverage increases prescription practice is aligned with the results of other studies and highlights the importance of health benefits and provincial formulary coverage. In 2011, Quebec became the first province in Canada to cover ticagrelor and prasugrel after an ACS; on discharge, Quebec showed increased use of ticagrelor and prasugrel (37.1%) compared to rest of Canada (19.2%) [12]. In 2008, Jackevicius et al. published on the impact of ODB coverage and clopidogrel use among patients who underwent PCI after an acute MI in Ontario; demonstrating an increase from 35% utilization before coverage to 88% after coverage in the first 30 days after discharge (*p*<0.001) [41]. Though adherence to guideline-directed use of DAPT following ACS will never reach 100%, targeted knowledge translation strategies are required to improve antiplatelet management for ACS patients in Ontario and around the world. As per the practical knowledge translation framework, a national survey to understand barriers towards appropriate antiplatelet prescription is required [42]. First, this would help address the underutilization of ticagrelor as compared to clopidogrel, and would aim to address the subset of ACS patients undergoing CABG, where surgeons are least adherent to prescribing guideline-directed DAPT. Increased prescription compliance in this population could close the practice gap and improve secondary prevention for these patients. Lastly, knowledge translation strategies should be developed during the medication study period (the pre-trial results stage) to expedite uptake if the study is positive.

### Limitations

Our study has several limitations. First, we included patients who had a coronary angiogram within 3 months after an ACS. Given this, there is a potential for survival bias in our cohort assembly since some patients may have died before an angiogram and may represent a population with different prescription patterns. While we discuss P2Y12 inhibitor prescription practices in our study, it should be noted that patient compliance is a significant limitation of our study. That is, our data capture is dependent on patients filling their prescriptions and not whether physicians prescribed the appropriate medication. Similarly, not all patients are compliant with over-the-counter ASA, and the inability to capture how many patients were taking this also remains a limitation. Last, 24.5% of medically managed patients, 20.7% of PCI patients, and 13.3% of CABG patients were on P2Y12 inhibitors before presenting with an ACS. Presumably, if these patients were continued on the same, they may not have required a prescription refill within the 14 days after discharge, leading to a miscount of actual P2Y12 inhibitor utilization. Lastly, a significant number of patients were on more potent anticoagulants (either a vitamin K antagonist or a direct oral anticoagulant) and may present a higher risk for bleeding, contraindicating the addition of a P2Y12 inhibitor, especially given the elderly age of our cohort.

Furthermore, the study design was such that it did not capture patients under the age of 65, nor capture prescriptions not covered by ODB. Due to this, we did not capture the prescription utilization of younger ACS patients. And we can only make assumptions about ticagrelor utilization rates prior to ODB coverage based on the decline demonstrated in utilization of other P2Y12 inhibitors. For instance, the drop in clopidogrel and all available P2Y12 inhibitor prescriptions within the month after ticagrelor’s Health Canada approval and the 2010 CCS Antiplatelet Therapy Guideline publication likely reflects an increase in out-of-pocket (paid-for by patients) ticagrelor prescriptions. Furthermore, the use of “Brilinta Savings Card,” which allowed some patients to pay as low as $5 for a 30 day supply of ticagrelor after Health Canada approval, along with samples from drug companies, most likely led to the decline in utilization of all available P2Y12 inhibitors, as more patients switched to ticagrelor. However, we cannot confirm this hypothesis.

## Conclusion

Our study shows an overall increase in utilization of all available P2Y12 inhibitors for elderly ACS patients compared to previous Canadian national registries. Government-led policy changes, such as public funding, appear to be most effective at moving clinicians towards guideline-directed medical therapy.

Compared to ACS patients who underwent CABG or medical management, patients who underwent PCI had a substantially greater increase in appropriate P2Y12 inhibitor utilization after implementation strategies. Future research must focus on the barriers to appropriate antiplatelet therapy in ACS patients, especially among those managed surgically.

## Data Availability

The data that support the findings of this study are available from ICES, but restrictions apply to the availability of these data, which were used under licensure for the current study, and so are not publicly available. Data are however available from the authors upon reasonable request and with permission of ICES. Note: ICES is an independent, non-profit research institute whose legal status under Ontario’s health information privacy law allows it to collect and analyze health care and demographic data, without consent, for health system evaluation and improvement.
